# Mental Health Challenges during the COVID-19 Pandemic

**DOI:** 10.3390/jcm12031213

**Published:** 2023-02-03

**Authors:** Alfonso Troisi

**Affiliations:** International Medical School, University of Rome Tor Vergata, via Montpellier 1, 00133 Rome, Italy; alfonso.troisi@uniroma2.it

The impact of the COVID-19 pandemic on mental health has unveiled the complexity of the relationship between psychiatry and the rest of medicine, as clearly shown by the collection of studies published in this Special Issue entitled “Mental Health Challenges during the COVID-19 Pandemic”.

Mental health and well-being depend on the combination of many individual variables, including genetic background, physiological homeostasis, child rearing experiences, socio-economic conditions, lifestyle, and interpersonal relationships. Although these variables are partly interconnected, they belong to distinct levels of analysis and are the object of study of different disciplines. It is uncommon to observe a single medical condition causing adverse effects on mental health through its concurrent impact on many of the variables listed above. Yet, this is exactly what has been happening with the COVID-19 pandemic.

The papers by Muñoz-Fernández et al. [1] and Kavvadas et al. [2] show that home confinement and alteration of daily activities were associated with increased levels of negative affectivity in Spanish adolescents and Greek university students, respectively. These findings are in line with growing evidence showing the importance of rewarding interpersonal relationships for establishing and maintaining optimal levels of psychological well-being [3]. This explains why preventive measures that proved to be effective in terms of controlling the spread of the virus led to other problems, particularly relating to the mental health of younger people.

Reviewing research and clinical data on the neurological effects of the SARS-CoV-2 infection, the papers by Zia et al. [4] and Ali Awan et al. [5] focused on the other extreme of the continuum that extends from the social to the organic. COVID-19 may be associated with a variety of neurologic complications, and several plausible mechanisms exist to account for these observations. As the current understanding of COVID-19 continues to evolve, a synthesis of the literature on the neurological impact of this novel virus may help inform clinical management and highlight potentially important avenues of investigation. Interestingly, the pathogenesis of neurological damage can involve indirect mechanisms, as shown by Rajagopalan et al. [6], who found alterations in fetal brainstem structure associated with increased maternal perception of pandemic-related stress in pregnant women.

Pregnant women belong to those special populations that have been responding to the challenge of the COVID-19 pandemic in peculiar ways, as shown by the review paper by Mazurchiewicz et al. [7]. Other special populations were investigated by Letica-Crepulja et al. [8], who analyzed symptom levels and coping strategies during the COVID-19 pandemic among treatment-seeking veterans with pre-existing post-traumatic disorder (PTSD), and by Marino et al. [9], who assessed the efficacy of a web-based remote training program in the management of behavioral disorders of children with autism spectrum disorders. In effect, one of the few positive aspects imposed by the COVID-19 pandemic is that telehealth has been rapidly deployed to help meet critical mental health needs.

Not only individual but also group variables may modulate the impact of the COVID-19 on mental health. Cultural differences should not be neglected when developing public health strategies to mitigate the adverse effects of stress, social isolation, fear, and uncertainty. Emodi-Perlam et al. [10] conducted a cross-sectional online survey and found major differences in personal worries about physical health, finances, and relations with relatives and friends of participants living in Canada, Israel, and Poland. These findings are consistent with one of the basic postulates of contemporary psychotraumatology: the individual response to stressors and traumatic events depends in part on the cultural context in which the person lives and the order and priority of ideological values [11].

The paper by Gesi et al. [12] reports on the number and characteristics of subjects accessing the emergency rooms for suicidal behavior in three Emergency Departments in Lombardy (Italy) before (2019) and during the first wave (2020) of the COVID-19 pandemic. The proportion of subjects accessing the Emergency Department for suicidality was significantly higher in 2020 than in 2019. Interestingly, during the pandemic, a greater proportion of subjects did not show any mental disorders and were psychotropic drug-free.

Five of the 16 papers published in this Special Issue focused on the mental health problems of healthcare workers (HCWs) working in COVID-19 healthcare facilities. HCWs face a high risk of contracting a potentially severe viral infection, as shown by mortality statistics. At the same time, compared to the general population, they have a better knowledge of infection risk factors and are consistently adopting preventive measures because of their professional duties. Thus, they are the ideal sample to study the interaction between emotional and rational psychological factors in modulating individual levels of fear of infection and its impact on mental health. Olaya et al. [13] carried out a systematic review and meta-analysis of the prevalence of depression among HCWs during the first wave of the COVID-19 pandemic. They found that almost half of the frontline HCWs showed increased levels of depression. The papers by Perego et al. [14] and Soto-Cámara et al. [15] identified several variables that act as risk factors for the development of depression and anxiety in HCWs (i.e., female gender, less work experience, lower levels of perceived social support, living with minors). Troisi et al. [16] found that personality was a significant predictor of fear of infection in HCWs working in a COVID-19 university hospital. Those participants who reported a more intense fear of infection had higher levels of neuroticism and fearful attachment. Considering that excessive fear of infection can put at risk HCWs’ psychological well-being and occupational efficiency, these findings can be useful to identify vulnerable subgroups and to implement selective programs of prevention based on counseling and psychological support. In their paper, Llorente-Alonso et al. [17] discuss the utility of selective programs such as job crafting and psychological empowerment to reduce the emotional distress of HCWs fighting the COVID-19 pandemic.

The burden of taking care of COVID-19 patients falls not only on HCWs, but also on their family caregivers. Apostol-Nicodemus et al. [18] carried out a cohort prospective study to assess the psychosocial impact of the pandemic on Philippine families of adult COVID-19 patients in isolation. They found that 43.2% of the caregivers had anxiety symptoms and 16.2% had depressive symptoms two weeks after the discharge of their relatives with a COVID-19 infection.

Before the COVID-19 pandemic, it was difficult to imagine that the worldwide spread of a respiratory infectious disease could have so many implications for mental health. The papers published in this Special Issue help us to understand why this has been happening. By definition, psychiatry is an interdisciplinary field extending from the investigation of neural correlates to the analysis of social dynamics [19]. The challenge of facing a global infectious threat has confirmed the importance of psychiatry for the rest of medicine and the necessity of considering the relationship between physical and mental health in terms of bidirectional pathways.

In this regard, one specific aspect is worth discussing. The COVID-19 pandemic has revealed how primitive emotional reactions to the risk of being infected by a potentially severe contagious disease can impact prevention and treatment programs.

There is a substantial difference between the fear of infection and the fear of the degenerative diseases that rank at the top of the morbidity statistics in affluent countries. Cancer, Alzheimer’s disease, heart disease, stroke, and diabetes are evolutionary novelties because their etiology and pathogenesis depend largely on risk factors and life habits that are typical of modern environments (e.g., extended longevity, high-calorie diet, sedentary lifestyle, obesity, smoking, drinking alcohol, pollution, etc.). Our ancestors living in the natural environment were not exposed to these risk factors and, therefore, they had an infinitesimal likelihood of getting cancer or developing senile dementia. Meanwhile, they had a very high likelihood of dying from an infection. From an evolutionary perspective, this means that infectious diseases have exerted strong selective pressures on human psychology and behavior.

Selection pressures have reinforced our defenses against infections by causing the evolution of a behavioral immune system that is separate from, and complementary to, the physiological immune system. The behavioral immune system includes a set of proactive mechanisms that inhibit contact with pathogens in the first place. These mechanisms offer a sort of psychological and behavioral prophylaxis against infection [20,21]. Like the physiological immune system, the behavioral immune system includes both detection and response mechanisms. When an external cue connoting infection risk (e.g., seeing another person with symptoms of infectious disease) is detected, it triggers a cascade of emotional and behavioral responses that minimize the infection risk (e.g., through social avoidance of people who appear to pose an infection risk). Fear of infection and pathogen disgust sensitivity are the two psychological mechanisms serving the adaptive function of the behavioral immune system [22].

Fear and disgust are deeply rooted in our emotional brain, and their activation can interfere with the implementation of public health strategies based on rational decisions. For example, one study reported a correlation between higher pathogen disgust sensitivity and negative attitudes toward COVID-19 vaccination [23]. A possible explanation for the negative impact of high pathogen disgust sensitivity on vaccination adherence is that vaccines are administered in ways that in and by themselves are cues to contamination, such as puncturing the skin or the inhalation or ingestion of a foreign substance [24].

One should consider that vaccination is an evolutionary novelty not directly linked with the cues that activate the behavioral immune system. Accordingly, the intention to vaccinate is a deliberate, conscious choice which might be only partially related to individual differences in germ aversion. In effect, when studies have focused on preventive measures other than vaccination, the functional utility of the behavioral immune system for combating the COVID-19 pandemic has emerged clearly. Shook et al. [25] found that germ aversion correlated with the frequency of preventive health behaviors such as social distancing, avoiding touching one’s face, wearing a facemask, hand washing and disinfecting objects. Cox et al. [26] reported that heightened disgust proneness before the pandemic resulted in an increased use of protective behaviors during the pandemic. Makhanova and Shepherd [27] found that germ aversion was negatively associated with the number of face-to-face interactions and positively associated with anxiety about social proximity.

The study of the behavioral immune system is a paradigmatic model for understanding the complex relationship between psychiatry and the rest of medicine. For example, there is evidence that, when social distancing results in social isolation, the functionality of the physiological immune system is reduced [28]. By contrast, the activity of the physiological immune system is enhanced by visual exposure to symptoms of infectious disease in others [29]. In conclusion, a lesson we are learning from the COVID-19 pandemic is that public health strategies should routinely include psychiatry and allied disciplines within the theoretical framework developed for optimizing prevention and treatment programs.

## References

[1] Muñoz-Fernández N., Rodríguez-Meirinhos A. (2021). Adolescents’ Concerns, Routines, Peer Activities, Frustration, and Optimism in the Time of COVID-19 Confinement in Spain. J. Clin. Med..

[2] Kavvadas D., Kavvada A., Karachrysafi S., Papaliagkas V., Cheristanidis S., Chatzidimitriou M., Papamitsou T. (2022). Stress, Anxiety and Depression Prevalence among Greek University Students during COVID-19 Pandemic: A Two-Year Survey. J. Clin. Med..

[3] Troisi A. (2020). Social stress and psychiatric disorders: Evolutionary reflections on debated questions. Neurosci. Biobehav. Rev..

[4] Zia N., Ravanfar P., Allahdadian S., Ghasemi M. (2022). Impact of COVID-19 on Neuropsychiatric Disorders. J. Clin. Med..

[5] Ali Awan H., Najmuddin Diwan M., Aamir A., Ali M., Di Giannantonio M., Ullah I., Shoib S., De Berardis D. (2021). SARS-CoV-2 and the Brain: What Do We Know about the Causality of ‘Cognitive COVID?. J. Clin. Med..

[6] Rajagopalan V., Reynolds W., Zepeda J., Lopez J., Ponrartana S., Wood J., Ceschin R., Panigrahy A. (2022). Impact of COVID-19 Related Maternal Stress on Fetal Brain Development: A Multimodal MRI Study. J. Clin. Med..

[7] Mazurkiewicz D., Strzelecka J., Piechocka D. (2022). Adverse Mental Health Sequelae of COVID-19 Pandemic in the Pregnant Population and Useful Implications for Clinical Practice. J. Clin. Med..

[8] Letica-Crepulja M., Stevanović A., Palaić D., Vidović I., Frančišković T. (2022). PTSD Symptoms and Coping with COVID-19 Pandemic among Treatment-Seeking Veterans: Prospective Cohort Study. J. Clin. Med..

[9] Marino F., Chilà P., Failla C., Minutoli R., Vetrano N., Luraschi C., Carrozza C., Leonardi E., Busà M., Genovese S. (2022). Psychological Interventions for Children with Autism during the COVID-19 Pandemic through a Remote Behavioral Skills Training Program. J. Clin. Med..

[10] Emodi-Perlman A., Eli I., Uziel N., Smardz J., Khehra A., Gilon E., Wieckiewicz G., Levin L., Wieckiewicz M. (2021). Public Concerns during the COVID-19 Lockdown: A Multicultural Cross-Sectional Study among Internet Survey Respondents in Three Countries. J. Clin. Med..

[11] Troisi A. (2018). Psychotraumatology: What researchers and clinicians can learn from an evolutionary perspective. Semin Cell Dev Biol..

[12] Gesi C., Grasso F., Dragogna F., Vercesi M., Paletta S., Politi P., Mencacci C., Cerveri G. (2021). How Did COVID-19 Affect Suicidality? Data from a Multicentric Study in Lombardy. J. Clin. Med..

[13] Olaya B., Pérez-Moreno M., Bueno-Notivol J., Gracia-García P., Lasheras I., Santabárbara J. (2021). Prevalence of Depression among Healthcare Workers during the COVID-19 Outbreak: A Systematic Review and Meta-Analysis. J. Clin. Med..

[14] Perego G., Cugnata F., Brombin C., Milano F., Preti E., Di Pierro R., De Panfilis C., Madeddu F., Di Mattei V. (2022). The “healthcare workers’ wellbeing [Benessere Operatori]” project: A longitudinal evaluation of psychological responses of Italian healthcare workers during the COVID-19 pandemic. J. Clin. Med..

[15] Soto-Cámara R., Navalpotro-Pascual S., Jiménez-Alegre J., García-Santa-Basilia N., Onrubia-Baticón H., Navalpotro-Pascual J., Thuissard I., Fernández-Domínguez J., Matellán-Hernández M., Pastor-Benito E. (2022). On behalf of the IMPSYCOVID-19 Study Group Influence of the Cumulative Incidence of COVID-19 Cases on the Mental Health of the Spanish Out-of-Hospital Professionals. J. Clin. Med..

[16] Troisi A., Nanni R., Riconi A., Carola V., Di Cave D. (2021). Fear of COVID-19 among Healthcare Workers: The Role of Neuroticism and Fearful Attachment. J. Clin. Med..

[17] Llorente-Alonso M., García-Ael C., Topa G., Sanz-Muñoz M., Muñoz-Alcalde I., Cortés-Abejer B. (2021). Can Psychological Empowerment Prevent Emotional Disorders in Presence of Fear of COVID-19 in Health Workers? A Cross-Sectional Validation Study. J. Clin. Med..

[18] Apostol-Nicodemus L., Tabios I., Limpoco A., Domingo G., Tantengco O. (2022). Psychosocial Distress among Family Members of COVID-19 Patients Admitted to Hospital and Isolation Facilities in the Philippines: A Prospective Cohort Study. J. Clin. Med..

[19] Troisi A. (2022). Biological psychiatry is dead, long live biological psychiatry!. Clin. Neuropsychiatry.

[20] Schaller M., Murray D.R., Bangerter A. (2015). Implications of the behavioural immune system for social behaviour and human health in the modern world. Philos. Trans. R. Soc. London. Ser. B Biol. Sci..

[21] Iwasa K., Yamada Y., Tanaka T. (2021). Editorial: Behavioral Immune System: Its Psychological Bases and Functions. Front. Psychol..

[22] Troisi A. (2020). Fear of COVID-19: Insights from Evolutionary Behavioral Science. Clin. Neuropsychiatry.

[23] Kempthorne J.C., Terrizzi J.A. (2021). The behavioral immune system and conservatism as predictors of disease-avoidant attitudes during the COVID-19 pandemic. Personal. Individ. Differ..

[24] Clay R. (2017). The Behavioral Immune System and Attitudes About Vaccines: Contamination Aversion Predicts More Negative Vaccine Attitudes. Soc. Psychol. Personal. Sci..

[25] Shook N.J., Sevi B., Lee J., Oosterhoff B., Fitzgerald H.N. (2020). Disease avoidance in the time of COVID-19: The behavioral immune system is associated with concern and preventative health behaviors. PLoS ONE.

[26] Cox R.C., Jessup S.C., Luber M.J., Olatunji B.O. (2020). Pre-pandemic disgust proneness predicts increased coronavirus anxiety and safety behaviors: Evidence for a diathesis-stress model. J. Anxiety Disord..

[27] Makhanova A., Shepherd M.A. (2020). Behavioral immune system linked to responses to the threat of COVID-19. Personal. Individ. Differ..

[28] Hawkley L.C., Cacioppo J.T. (2010). Loneliness matters: A theoretical and empirical review of consequences and mechanisms. Ann. Behav. Med. A Publ. Soc. Behav. Med..

[29] Schaller M., Miller G.E., Gervais W.M., Yager S., Chen E. (2010). Mere visual perception of other people’s disease symptoms facilitates a more aggressive immune response. Psychol. Sci..

